# On a Closer Look of a Doppler Tolerant Noise Radar Waveform in Surveillance Applications †

**DOI:** 10.3390/s24082532

**Published:** 2024-04-15

**Authors:** Maximiliano Barbosa, Leandro Pralon, Antonio L. L. Ramos, José Antonio Apolinário

**Affiliations:** 1 Brazilian Navy Weapons Systems Directorate, Rio de Janeiro 20010-000, Brazil; 2 Brazilian Army Technological Center, Rio de Janeiro 23020-470, Brazil; pralon.leandro@eb.mil.br; 3 Department of Science and Industry Systems, University of South-Eastern Norway (USN), 3616 Kongsberg, Norway; antonior@usn.no; 4 Military Institute of Engineering, Rio de Janeiro 22290-270, Brazil; apolin@ime.eb.br

**Keywords:** noise radar, APCN, electronic support measures, time-frequency analysis

## Abstract

The prevalence of Low Probability of Interception (LPI) and Low Probability of Exploitation (LPE) radars in contemporary Electronic Warfare (EW) presents an ongoing challenge to defense mechanisms, compelling constant advances in protective strategies. Noise radars are examples of LPI and LPE systems that gained substantial prominence in the past decade despite exhibiting a common drawback of limited Doppler tolerance. The Advanced Pulse Compression Noise (APCN) waveform is a stochastic radar signal proposed to amalgamate the LPI and LPE attributes of a random waveform with the Doppler tolerance feature inherent to a linear frequency modulation. In the present work, we derive closed-form expressions describing the APCN signal’s ambiguity function and spectral containment that allow for a proper analysis of its detection performance and ability to remove range ambiguities as a function of its stochastic parameters. This paper also presents a more detailed address of the LPI/LPE characteristic of APCN signals claimed in previous works. We show that sophisticated Electronic Intelligence (ELINT) systems that employ Time Frequency Analysis (TFA) and image processing methods may intercept APCN and estimate important parameters of APCN waveforms, such as bandwidth, operating frequency, time duration, and pulse repetition interval. We also present a method designed to intercept and exploit the unique characteristics of the APCN waveform. Its performance is evaluated based on the probability of such an ELINT system detecting an APCN radar signal as a function of the Signal-to-Noise Ratio (SNR) in the ELINT system. We evaluated the accuracy and precision of the random variables characterizing the proposed estimators as a function of the SNR. Results indicate a probability of detection close to 1 and show good performance, even for scenarios with a SNR slightly less than −10 dB. The contributions in this work offer enhancements to noise radar capabilities while facilitating improvements in ESM systems.

## 1. Introduction

Electronic Intelligence systems for Electronic Warfare rely on the detection, identification, and further processing of radar signals [1]. The advances in the semiconductor industry over the last decade enabled huge breakthroughs in ELINT systems. Indeed, modern equipment can implement high-frequency, high-bandwidth digital receivers with complex signal processing algorithms. An example is jammers capable of reproducing radar signal characteristics after extracting them. The design of Low Probability of Interception and Low Probability of Exploitation radars has attracted significant attention in the current technological race. As a result, the transmission of random or pseudo-random signals has gained considerable notoriety within the radar system community over the last few years [2,3,4,5,6,7,8,9,10]. Commonly known as “noise radars”, they manage to achieve LPI by employing pulse compression in the reception, along with a proper choice of transmitted waveform stochastic properties [2,11], LPE [9,12,13], and low sidelobe levels, while suppressing range ambiguity.

Many different approaches have been proposed in the literature to generate waveforms characterized by stochastic processes that better fulfill these radar system requirements [5,14,15,16,17,18]. Still, they all suffer from low Doppler tolerance [19], an inherent characteristic of traditional noise radars that hinders their use in several applications, including surveillance. Researchers have proposed alternative approaches to bypass such a weakness while preserving the LPI and the LPE properties of random waveforms. Included in this group is the so-called Advanced Pulse Compression Noise (APCN) waveform [3,20,21], which combines a random signal with a Doppler-tolerant Linear Frequency Modulation (LFM) waveform. The random signal may consist of amplitude terms, phase terms, or both.

In [22], we expanded the discussion over the performance of APCN waveforms. We derived closed-form expressions to characterize the APCN’s narrowband ambiguity function, the power spectral density, and the spectral containment, thus accounting for random components in the amplitude and phase of the transmitted signal. Furthermore, we identified and addressed a significant drawback in employing the APCN signal for detecting slow-moving targets. In the present work, we extend the results and findings of [22] by investigating the same properties for APCN signals with Phase-Only random components. Furthermore, we examine APCN’s exploitation of power transmission through the Peak-to-Average Power Ratio, its ability to mitigate range ambiguities, detect uncooperative targets, and determine their range and radial velocity in real-time. These factors are crucial in radar waveform design, particularly in surveillance applications.

From the perspective of an ESM system, as discussed in [20], employing an APCN signal with a particular level of phase randomness and constant amplitude introduces a challenge in accurately discerning the waveform characteristics for an intercept-receiver system. However, owing to significant recent advancements in emitter detection and exploitation techniques, modern ESM systems and traditional spectral analysis methods can generate Time-Frequency Analysis maps, as mentioned in [6,11,23]. As a result, it has become feasible to ascertain the waveform characteristics of deterministic LPI/LPE radar signal modulations.

References [24,25,26,27,28] introduce techniques for conducting TFA and extracting modulation parameters from deterministic signals assumed LPI/LPE. However, these techniques have a drawback: the extraction process relies exclusively on visual analysis. This dependency on human interpretation of TFA results hampers the effectiveness of non-real-time EW receivers. In contrast, the works in [29,30,31] propose autonomous extraction methods, albeit still primarily designed for deterministic signals. Nonetheless, a significant contribution of these works is in developing a technique that leverages image processing methodologies to autonomously extract characteristics of a noise radar that employs APCN, allegedly LPI, as discussed in [3,20]. Our analysis, however, considers varied signal-to-noise ratio levels in an ESM receiver chain to ensure robust and satisfactory results. In addition, it examines APCN waveforms featuring phase and amplitude random components, in contrast to the approach in [3] that focuses exclusively on phase randomness. Indeed, the extended configuration augments the level of randomness in APCN waveforms and enhances their LPI and LPE characteristics, as corroborated by the authors of the present work in [22].

The rest of this paper unfolds as follows. Section 2 explores noise radar operations focusing on the APCN waveform as the transmitting signal in surveillance applications. Section 3 delves into the modeling of an ESM digital system, offering an in-depth analysis of the APCN radar waveform from the perspective of an intercept–receiver system. Moreover, the section introduces a methodology designed to detect and extract the distinctive characteristics of APCN signals. It also includes a comprehensive discussion of the performance evaluations of this methodology, laying out the grounds for conclusions regarding the LPI and LPE potential of the APCN waveform in an EW scenario. Section 4 presents the conclusions and summarizes the key findings and insights in this paper.

## 2. Advanced Pulse Compression Noise Radars

In traditional noise radar systems, the transmit signal is characterized by a stochastic process, s(t) [2]. Consequently, the corresponding matched filter outputs, relative to the pulse compression architectures, are all characterized by complex random processes. Therefore, a proper analysis of noise radars must rely on probabilistic tools. That is the case of the narrowband ambiguity function of a random signal, given as [32]

(1) $$\chi_{\tilde{s}}\left(\tau ,f_{D}\right)=\int_{-\infty }^{\infty }\tilde{s}\left(t\right)\;{\tilde{s}}^{*}\left(t-\tau \right)\;e^{-j2\pi f_{D}t}dt,$$

where $\tilde{s}\left(t\right)$ is the complex envelope of s(t), $f_{D}$ is the Doppler frequency, and τ is the time delay. Assuming $\tilde{s}\left(t\right)$ to be Wide-Sense Stationary (WSS) and time-limited with duration $\tau_{s}$, and that $f_{D}$ is deterministic, the expected value of the ambiguity function of Equation (1) simplifies to [5,19]

(2) $$E\left[\chi_{\tilde{s}}\left(\tau ,f_{D}\right)\right]=\tau_{s}\;R_{\tilde{s}}\left(\tau \right)\;\mathrm{sinc}\left(f_{D}\tau_{s}\right);\;\;\;\;\;-\tau_{s}\leq \tau \leq \tau_{s},$$

where $R_{\tilde{s}}\left(\tau \right)=E\left[\tilde{s}\left(t\right)\;{\tilde{s}}^{*}\left(t-\tau \right)\right]$ is the autocorrelation function of the complex stochastic process $\tilde{s}\left(t\right)$ [33].

The near thumbtack format depicted in Equation (2) shows that the expected range and Doppler profiles in noise radar systems are independent functions; therefore, no range-Doppler coupling is expected in noise radars, inhibiting their usage in surveillance applications, for example [34].

To overcome the no range-Doppler coupling limitation while preserving the random nature of the transmit signal, the authors in [3,20,21] proposed the Advanced Pulse Compression Noise (APCN) radar architecture. APCN waveforms combine a deterministic Linear Frequency Modulation signal with a stochastic component. Its complex envelope is given by [20,22]

(3) $$\tilde{s}\left(t\right)=\sqrt{P}\;a\left(t\right)\;e^{j[\theta (t)+\kappa \;\phi (t)]},$$

where the samples of the random process a(t) have a Rayleigh distribution, i.e., $p\left(a\right)=\left(a/\alpha^{2}\right)\;e^{-a^{2}/\alpha^{2}}$, with a≥0 and α being the scale parameter. Moreover, ϕ(t) is uniformly distributed in the interval (0,2π] and θ(t) represents the LFM deterministic component. The signal’s complex envelope mean power, *P*, is assumed, with no loss of generality, to be unitary throughout the present work. We can rewrite Equation (3) as $\tilde{s}\left(t\right)={\tilde{s}}_{r}\left(t\right)\;{\tilde{s}}_{c}\left(t\right)$, where ${\tilde{s}}_{r}\left(t\right)=a\left(t\right)\;e^{j\kappa \phi (t)}$ is the transmit signal random component, with 0≤κ≤1, and ${\tilde{s}}_{c}\left(t\right)=e^{j\theta (t)}$ is the LFM waveform envelope, with bandwidth $\beta_{{\tilde{s}}_{c}}$.

In modern radar systems, the matched filter is implemented digitally. Therefore, we consider the discrete-time case in our analysis and define

$$\tilde{s}\left(n\right)=\tilde{s}{\left(t\right)}_{{|}_{t=nT}},$$

where *n* is the discrete-time index and $T=1/f_{s}$, with $f_{s}$ being the sampling frequency. We also assume that $\tau =\bar{\tau }T$, where $\bar{\tau }$ corresponds to the discrete-time delay in samples. Moreover, $\tau_{s}={\bar{\tau }}_{s}T$, with $\bar{\tau_{s}}$ being the number of samples in a pulse signal with duration $\tau_{s}$.

We show in [22] that the APCN narrowband ambiguity function is such that

(4) $$E\left[\chi_{\tilde{s}}\left(\bar{\tau },f_{D}\right)\right]=R_{{\tilde{s}}_{r}}\left(\bar{\tau }\right)\;\chi_{{\tilde{s}}_{c}}\left(\bar{\tau },f_{D}\right);\;\;\;\;\;\bar{\tau }\leq \left|{\bar{\tau }}_{s}\right|,$$

where $R_{{\tilde{s}}_{r}}\left(\bar{\tau }\right)$ is the autocorrelation sequence of the transmit signal random component given as

(5) $$R_{{\tilde{s}}_{r}}\left(\bar{\tau }\right)=\alpha^{2}\left(\frac{1-\cos2\kappa \pi }{4\kappa^{2}\pi }\right)+\left[2\alpha^{2}-\alpha^{2}\left(\frac{1-\cos2\kappa \pi }{4\kappa^{2}\pi }\right)\right]\delta \left(\bar{\tau }\right),$$

where $\delta (\bar{\tau })$ is the Dirac delta sequence and $\chi_{{\tilde{s}}_{c}}\left(\bar{\tau },f_{D}\right)$ in (4) is the ambiguity function of ${\tilde{s}}_{c}\left(t\right)$ defined as

(6) $$\begin{array}{c} \chi_{{\tilde{s}}_{c}}\left(\bar{\tau },f_{D}\right)=\left(1-\frac{|\bar{\tau }|}{{\bar{\tau }}_{s}}\right)\left|\frac{\sin\left[\pi \left(\frac{f_{D}}{f_{s}}+\frac{\mu }{f_{s}^{2}}\bar{\tau }\right)({\bar{\tau }}_{s}-|\bar{\tau }\left|\right)\right]}{\pi \left(\frac{f_{D}}{f_{s}}+\frac{\mu }{f_{s}^{2}}\bar{\tau }\right)({\bar{\tau }}_{s}-|\bar{\tau }\left|\right)}\right|,\;\bar{\tau }\leq \left|{\bar{\tau }}_{s}\right|\;, \end{array}$$

wherein $\mu =\beta_{{\tilde{s}}_{c}}/\tau_{s}$.

**Figure 1:**
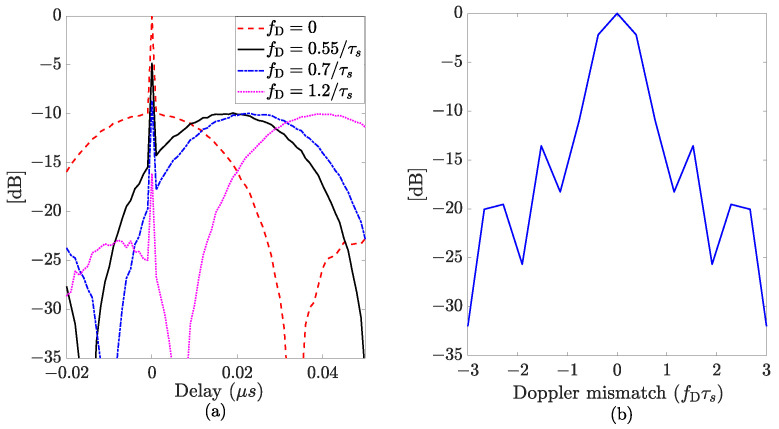
Normalized ambiguity function (in dB) considering a single pulse realization of an APCN waveform (κ=0.5 and α=1): (**a**) range profile (Doppler cuts); (**b**) Doppler profile (zero-delay cut).

Figure 1a illustrates the range profile for different values of Doppler shifts, namely $f_{D}=0$, $f_{D}=0.55/\tau_{s}$, $f_{D}=0.7/\tau_{s}$, and $f_{D}=1.2/\tau_{s}$, of an APCN normalized ambiguity function, with κ=0.5 and α=1, and considering $\beta_{{\tilde{s}}_{c}}=30$ MHz and $\tau_{s}=50$ μs. The sampling frequency was assumed to be $f_{s}=1$ GHz. Figure 1b shows the zero-delay cut behavior (Doppler profile).

Despite being Doppler tolerant, APCN waveforms exhibit an anomaly at zero delays, potentially impairing radar performance in the presence of slow-moving targets. A closer inspection of Equations (4)–(6) evidences the presence of a sinc function at the pulse compression output whose maximum peak exists at $f_{D}/fs+\mu \;\bar{\tau }/f_{s}^{2}=0$. The sinc function is attenuated by a factor $L=H\;(1-f_{D}/\beta_{\tilde{s_{c}}})$, for $\bar{\tau }\neq 0$, and amplified by $A=2\alpha^{2}$, for $\bar{\tau }=0$, with $H=\alpha^{2}\left[1-\cos(2\kappa \pi \right)]/4\kappa^{2}\pi$, as a result of Equation (5). Additionally, and analogously to the LFM case, it presents a range measurement error $\epsilon_{R}=-cf_{D}\;\tau_{s}/2\beta_{\tilde{s_{c}}}$, that can be eliminated [34].

**Figure 2:**
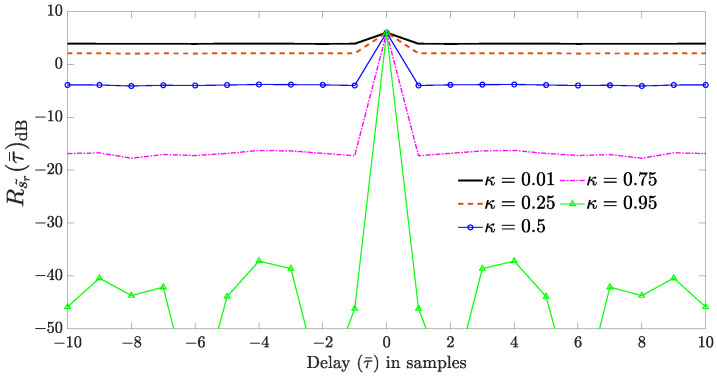
Autocorrelation sequence of the APCN complex random component for different values of κ, considering α=1.

Figure 2 illustrates one realization of the APCN random component autocorrelation sequence (see Equation (5)), considering a signal ${\tilde{s}}_{r}\left(n\right)$ composed of a random amplitude with scale parameter α=1 and a random phase uniformly distributed in the interval (0,2κπ], for different values of κ. Here, we can see that the attenuation on the APCN range profile increases with κ for $\bar{\tau }\neq 0$. The implication is that when a target exhibits a Doppler shift the peak of the range profile shifts away from the origin because of the Doppler-tolerant behavior of the LFM component, making it susceptible to attenuation. Indeed, this behavior reduces the SNR, diminishing the system’s Probability of Detection.

The Dirac delta component in Equation (5) is always present for $\bar{\tau }=0$. For slow-moving targets, i.e., those that give rise to a Doppler shift above a threshold greater than $0.55/\tau_{s}$, in this specific case, the spike falls outside the range resolution, given by the sinc function main lobe’s 3 dB width, but still exhibits enough power to pose as another target’s response. Thus, the contribution of the noisy component to the matched filter output can lead to target misdetection or false alarms due to misinterpretations.

**Figure 3:**
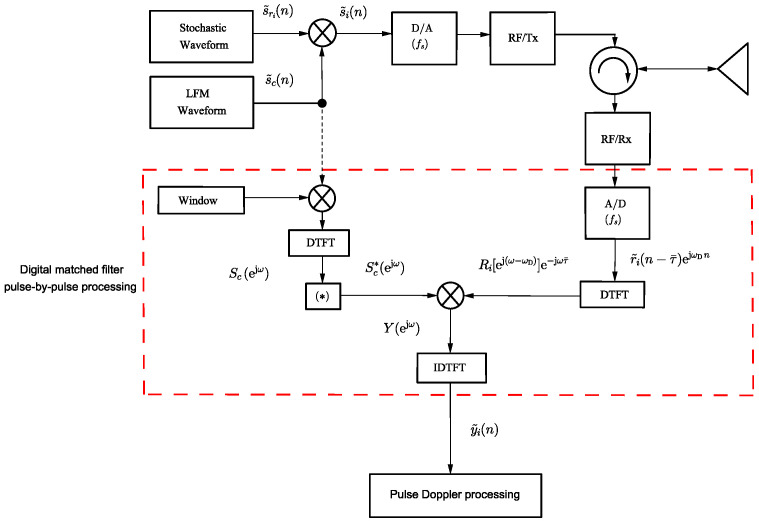
Proposed simplified radar block diagram to obtain the matched filter output pulse-by-pulse, where (*) is the complex conjugate operation.

In [22], the authors proposed a method to eliminate the spike at $\bar{\tau }=0$ by correlating the received signal with the deterministic component of the APCN waveform instead of using the transmit signal’s replica in a pulse compression architecture. Figure 3 presents a simplified block diagram of the proposed system implemented in a digital receiver signal processing chain. This modification makes it possible to eliminate the impulse anomaly at $\bar{\tau }=0$, leaving only the deterministic component attenuation $\left(1-f_{D}/\beta_{\tilde{s_{c}}}\right)$ due to the Doppler mismatch. In this diagram, index “*i*” represents the *i*-th generated sample function of a stochastic process in the transmission of the *i*-th pulse, and $\omega_{D}=2\pi f_{D}/f_{s}$ is the digital Doppler frequency. We adopted the Hann window to weigh the replica due to its demonstrated advantages in minimizing the integrated side-lobe ratio, which favors range resolution [21].

In light of the physics governing the phenomenon, for simplification and without sacrificing generality, let us consider a received signal $\tilde{r}\left(t\right)=\tilde{s}\left(t-T_{0}\right)e^{-j2\pi f_{D}t}$ from a scatter located at $R_{0}=c/2T_{0}$, with *c* being the vacuum light speed. The expected value of the pulse compression output resulting from the proposed architecture [22] (see Figure 3) is thus given by

(7) $$E\left[\tilde{y}\left(\tau \right)\right]=E\left[\int_{-\infty }^{\infty }\left[{\tilde{s}}_{r}\left(t-T_{0}\right){\tilde{s}}_{c}\left(t-T_{0}\right)\;e^{-j2\pi f_{D}t}\right]\;{\tilde{s}}_{c}^{*}\left(t-\tau \right)dt\right].$$

Since a(t) and ϕ(t) are WSS and independent process [20], Equation (7) can then be written as [33]

(8) $$E\left[\tilde{y}\left(\tau \right)\right]=E\left[a\left(t\right)\right]E\left[e^{j\kappa \phi (t)}\right]\;\chi_{{\tilde{s}}_{c}}\left(\tau -T_{0},f_{D}\right);\;\;\;\;T_{0}-\tau_{s}\leq \tau \leq T_{0}+\tau_{s},$$

which, in turn, can be shown to reduce to

(9) $$E\left[\tilde{y}\left(\tau \right)\right]=\alpha \sqrt{\frac{\pi }{2}}\left(\frac{\sin\kappa \pi }{\kappa \pi }\right)\;\chi_{{\tilde{s}}_{c}}\left(\tau -T_{0},f_{D}\right);\;\;\;\;T_{0}-\tau_{s}\leq \tau \leq T_{0}+\tau_{s}.$$

**Figure 4:**
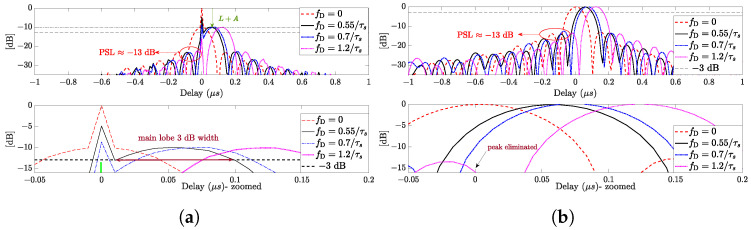
Pulse compression output for targets with different Doppler shifts: (**a**) considering the receiver system configuration based on filtering the received APCN signal with a transmit replica; (**b**) considering the receiver system using our proposal configuration.

As stated in Equation (9), the output of our proposed architecture [22] presents the shape of the sinc function relative to the LFM matched filter component, attenuated by a factor $B=\alpha \sqrt{\frac{\pi }{2}}\left(\frac{\sin\kappa \pi }{\kappa \pi }\right)$. Therefore, the pulse compression gain is compromised, and the proposed technique becomes effective only when the chosen APCN random component parameters are such that *B* is not so high as to start jeopardizing the SNR at the detector. Figure 4a, illustrates the normalized matched filter output of a conventional pulse compression radar architecture based on filtering the received APCN signal (α=1 and κ=0.5) with a transmit replica (proposed in [21]). Note the attenuation of the normalized sinc main lobe, given by L+A, for $\bar{\tau }\neq 0$, as derived previously. Note further that, since the attenuation is near the same for all $\bar{\tau }\neq 0$, the peak side-lobe (PSL) ratio achieved with the LFM signal is preserved and remains close to 13 dB [34]. These observations demonstrate that APCN signals do not present a PSL ratio close to the well-known time-bandwidth product inherent to traditional noise radar waveforms.

Figure 4b illustrates the normalized pulse compression output of our proposed architecture, considering targets with the same Doppler shifts as in Figure 4a. Note that the anomaly was eliminated for all analyzed Doppler shifts, minimizing the possibility of false targets. The PSL ratio also remained close to 13 dB.

The stochastic component a(t) in Equation (3) increases the transmit signal’s degree of randomness, potentially enhancing its LPI and LPE properties. Nevertheless, it introduces a significant drawback to the system related to the limited exploitation of the power transmitter. Such a drawback is evident in the reduction in the the Peak-to-Average Power Ratio (PAPR) of the system, given by [6,35]

(10) $$\mathrm{PAPR}=\frac{\max_{\left\{n\right\}}|{\tilde{s}\left(n\right)|}^{2}}{\frac{1}{{\bar{\tau }}_{s}}\sum_{n=1}^{{\bar{\tau }}_{s}}{\left|\tilde{s}\left(n\right)\right|}^{2}}.$$

Signals characterized by a consistent amplitude envelope exhibit a PAPR of 1, rendering them highly power-efficient. These signals enable the driving of power amplifiers close to saturation, maximizing power utilization during transmission. Any departure from the unity amplitude level results in an energy loss within the correlation main lobe, ultimately reducing the peak response level. The energy loss can be interpreted as an SNR degradation, leading to a decline in detection performance. Define the unavoidable decrease in performance as [6,13]

(11) $${\mathrm{SNR}}_{Loss}=-10\log_{10}\left(\mathrm{PAPR}\right).$$

As an alternative to amplitude modulation, the community has devoted significant effort to deriving closed-form expressions of random frequency-modulated signals for radar applications better suited for systems requiring high power efficiency. Assuming ${\tilde{s}}_{r}^{PO}\left(t\right)=e^{j\kappa \phi (t)}$, the autocorrelation sequence of the random component of the transmit Phase-Only (PO) APCN signal is given by [22]

(12) $$R_{{\tilde{s}}_{r}^{PO}}\left(\bar{\tau }\right)=\frac{1-\cos2\kappa \pi }{2\kappa^{2}\pi^{2}}+\left(\;1-\frac{1-\cos2\kappa \pi }{2\kappa^{2}\pi^{2}}\right)\delta \left(\bar{\tau }\right).$$

**Figure 5:**
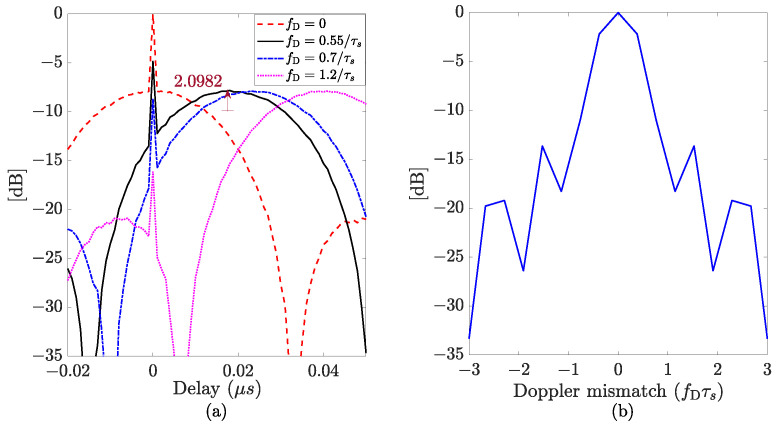
Normalized ambiguity (in dB) function considering a single pulse realization of a Phase-Only APCN waveform (κ=0.5): (**a**) range profile (Doppler cuts); (**b**) Doppler profile (zero-delay cut).

Analogously to Figure 1a, Figure 5a illustrates the Phase-Only APCN normalized range profile, considering different values of Doppler shifts ($f_{D}=0$, $f_{D}=0.55/\tau_{s}$, $f_{D}=0.7/\tau_{s}$, and $f_{D}=1.2/\tau_{s}$), κ=0.5, $\beta_{{\tilde{s}}_{c}}=30$ MHz, and $\tau_{s}=50\;\mu s$. The sampling frequency was also assumed to be $f_{s}=1$ GHz. Figure 5b shows the zero-delay cut behavior, nearly the same as in Figure 1b.

**Figure 6:**
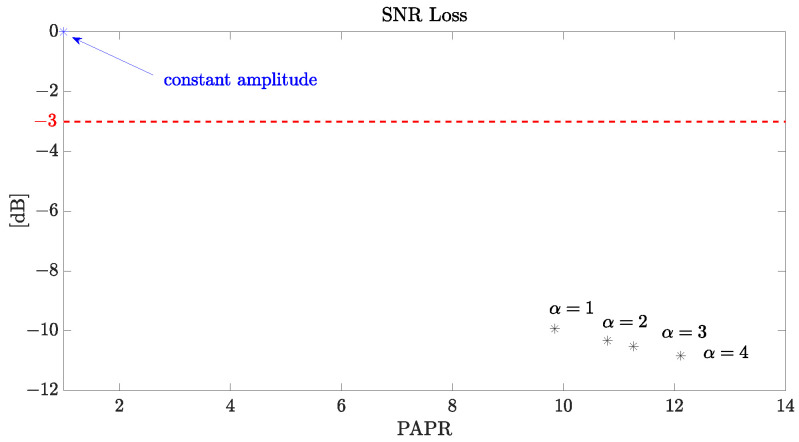
SNR loss versus PAPR assuming κ=0.5 and different scale parameters α.

In Figure 5a, it is noteworthy that the power level difference between the zero-delay (τ=0 μs) spike and the main lobe of the sync function is reduced by a factor of π/4 compared to Figure 1a. When converted to dB, this difference becomes $20\log_{10}\left(\pi /4\right)=2.0982\;$ dB. The reduction helps mitigate the adverse effects of such anomalies on the system’s detection performance. Additionally, Figure 6 illustrates the relationship between ${\mathrm{SNR}}_{\mathrm{Loss}}$ and various PAPR values, considering different scale parameters α associated with randomness in amplitude. A value of κ=0.5 is assumed. Notably, the Phase-Only (constant amplitude) APCN waveform achieves a PAPR < 2, which is acceptable in noise radar applications without substantial degradation in detection performance (${\mathrm{SNR}}_{Loss}$ < 3 dB) [6]. Furthermore, according to [13], when the random amplitude is employed, the resultant noisy waveform has a PAPR of around 10 (or greater), with reduced transmitted energy. 

Another property that we evaluated in dealing with random transmit signals is related to its spectral containment, which usually is not efficient. The analysis of the transmit signal’s Power Spectral Density (PSD), $\Gamma_{\tilde{s}}\left(\omega \right)$, given by the Discrete-Time Fourier Transform (DTFT) of its autocorrelation sequence, $R_{\tilde{s}}\left(\bar{\tau }\right)=E\left[\chi_{\tilde{s}}\left(\bar{\tau },0\right)\right]$ [33,36], allows for the evaluation of its spectral containment and the derivation of closed-form expressions describing its bandwidth. Considering Equation (5), the APCN PSD is given by

(13) $$\Gamma_{\tilde{s}}\left(\omega \right)=F\left\{H\;\chi_{{\tilde{s}}_{c}}\left(\bar{\tau },0\right)\right\}+\left(2\alpha^{2}-H\right)\;F\left\{\delta \left(\bar{\tau }\right)\right\}*\Gamma_{{\tilde{s}}_{c}}\left(\omega \right),$$

where * and F{·} are, respectively, the convolution and DTFT operators, $H=\alpha^{2}\left[1-\cos(2\kappa \pi \right)]/4\kappa^{2}\pi$, as previously defined, and $\Gamma_{{\tilde{s}}_{c}}\left(\omega \right)$ is the PSD of $R_{{\tilde{s}}_{c}}\left(\bar{\tau }\right)=\chi_{{\tilde{s}}_{c}}\left(\bar{\tau },0\right)$.

Expanding Equation (13) and knowing that $F\left\{\delta (n)\right\}=1$ yields

(14) $$\Gamma_{\tilde{s}}\left(\omega \right)=H\;\Gamma_{{\tilde{s}}_{c}}\left(\omega \right)+\left(2\alpha^{2}-H\right)\underset{1}{\underset{︸}{\frac{1}{2\pi }\int_{-\pi }^{\pi }\Gamma_{{\tilde{s}}_{c}}\left(\varsigma \right)\;d\varsigma }}.$$

Note that the integral on the right-hand side represents, in the digital frequency domain (−π≤ω≤π), the periodic sum of the samples of a complex envelope linear chirp signal with unitary power. Consequently, according to Parseval’s Theorem [36], it evaluates to one. Finally, Equation (14) becomes

(15) $$\Gamma_{\tilde{s}}\left(\omega \right)=H\;\Gamma_{{\tilde{s}}_{c}}\left(\omega \right)+\left(2\alpha^{2}-H\right),\left|\omega \right|\leq \pi .$$

From the closed-form transmits signal’s PSD in (15), there are two widespread approaches to define a signal’s bandwidth: the 3 dB bandwidth ($\beta_{3\mathrm{dB}}$) and the portion of the spectrum where p% of the total power is concentrated ($\beta_{p\%}$) [5]. Concerning the former and according to Equation (15), $\beta_{3\mathrm{dB}}$ can be considered the same $\beta_{{\tilde{s}}_{c}}$ of the APCN signal deterministic LFM component [3]. Nevertheless, the percentage of the total power within $\beta_{{\tilde{s}}_{c}}$ reduces as κ increases, leading to the rise of $\beta_{p\%}$, which can compromise the APCN waveform’s performance in practical applications.

The percentage of total power within $\beta_{\tilde{s}}$ is given by the integral of $\Gamma_{\tilde{s}}\left(\omega \right)$ in Equation (15) over the interval $\left[-\pi \beta_{{\tilde{s}}_{c}}/f_{s},\pi \beta_{{\tilde{s}}_{c}}/f_{s}\right]$; hence

(16) $$\begin{array}{cc} \%P_{\beta_{\tilde{s}}} & =\;\;\;\int_{-\pi \beta_{{\tilde{s}}_{c}}/f_{s}}^{\pi \beta_{{\tilde{s}}_{c}}/f_{s}}\;\left[H\;\Gamma_{{\tilde{s}}_{c}}\left(\omega \right)+\left(2\alpha^{2}-H\right)\right]d\omega \\ & =H\left[\int_{-\pi \beta_{{\tilde{s}}_{c}}/f_{s}}^{\pi \beta_{{\tilde{s}}_{c}}/f_{s}}\Gamma_{{\tilde{s}}_{c}}\left(\omega \right)\;d\omega \right]+\left(2\alpha^{2}-H\right)\frac{2\pi \beta_{{\tilde{s}}_{c}}}{f_{s}}. \end{array}$$

Practical situations require a higher $\beta_{{\tilde{s}}_{c}}\tau_{s}$ product to well-define the spectrum of an LFM waveform [34]. Owing to the maximum total power contained within a rectangular bandwidth shape, there is minimal spreading of the LFM spectrum. Consequently, the integral in Equation (16) can be approximated as one, simplifying to

(17) $$\%P_{\beta_{\tilde{s}}}=H+2\pi \left(2\alpha^{2}-H\right)\;\frac{\beta_{{\tilde{s}}_{c}}}{f_{s}}.$$

In turn, $\beta_{p\%}$ can be shown as in

(18) $$\beta_{p\%}=\frac{\left(p-H\right)f_{s}}{2\pi (2\alpha^{2}-H)},$$

where *p* is the desired percentage power within $\beta_{\tilde{s}}$.

A similar derivation applies to the Phase-Only APCN waveform. Analogously to the previous formulation, one can show that the percentage of total power within $\beta_{\tilde{s}}$ when the APCN signal’s amplitude is constant is given by

(19) $$\%P_{\beta_{\tilde{s}}}^{PO}=D+2\pi \left(1-D\right)\;\frac{\beta_{{\tilde{s}}_{c}}}{f_{s}},$$

where $D=\left[1-\cos(2\kappa \pi \right)]/2\kappa^{2}\pi^{2}$. Thus, it be shown that $\beta_{p\%}$ becomes

(20) $$\beta_{p\%}^{PO}=\frac{\left(p-D\right)f_{s}}{2\pi (1-D)},$$

where *p* is the desired percentage power within $\beta_{\tilde{s}}$.

**Figure 7:**
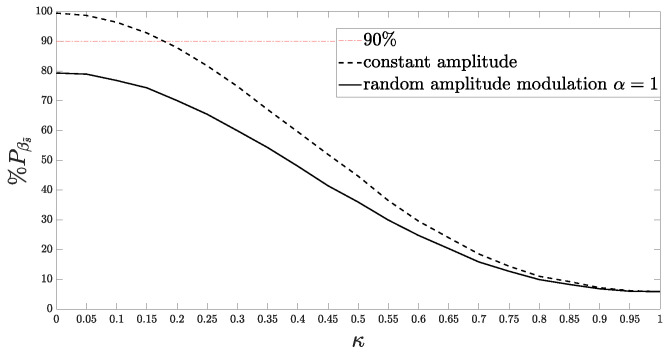
Relationship between % of total power in bandwidth and κ, assuming constant and random amplitude (α=1) modulation.

Figure 7 presents the behavior of $\%P_{\beta_{\tilde{s}}}$ for different values of κ, assuming constant and random discrete amplitude a(n). Note that the variability in phase randomness directly impacts the mean power of the APCN transmitted signal within the desired bandwidth. Moreover, introducing randomness in amplitude creates a greater challenge in maintaining the transmit waveform spectral confinement. Consequently, the RF transmitter, receiver, and the entire signal processing chain must account for these effects to prevent signal distortions or a misformulation of the radar range equation, ultimately leading to a degradation in detection performance.

As a rule of thumb, radar system design good practice recommends that the percentage of power within the designated bandwidth should be close to 90% [5]. Nevertheless, this criterion is met only for low values of κ regardless of whether or not one considers random amplitude. On the other hand, the value of κ directly influences the APCN signal’s degree of randomness and, consequently, its LPI/LPE performance [20] as well. Specifically, the greater the value of κ, the more random the resultant APCN signal should be. Therefore, one must evaluate the LPI/LPE performance as a function of the stochastic component parameters of APCN waveforms to establish proper trade-offs during design.

The noise radar system LPI/LPE characteristics are twofold. Firstly, these systems can effectively mimic thermal noise, rendering them indistinguishable from less sophisticated ELINT systems and ensuring covert operation. Secondly, they can produce varied sample functions from the same stochastic process. This capability diminishes the effectiveness of deception systems that do not operate in real time. Many works in the literature, e.g., [4,5,6,9,13], attempt to define proper metrics to evaluate a system’s LPI/LPE characteristic properly. Despite the different approaches, a common sense within the community is that these features relate to the transmitted waveform degree of randomness.

In [4,5,9,13], for instance, the authors propose the analysis of the transmitted signal’s Spectral Flatness Measure (SFM) and Mutual Information Rate (MIR) as measures of its information increase with time. For Gaussian processes [37], the SFM is directly related to the MIR and is defined as the ratio of the geometric mean to the arithmetic mean of the signal’s PSD, given by [37]

(21) $$\mathrm{SFM}=\frac{\exp\left[\frac{1}{2\pi }\int_{-\pi }^{\pi }\ln(\Gamma_{\tilde{s}}\left(\omega \right))\;d\omega \right]}{\frac{1}{2\pi }\int_{-\pi }^{\pi }\Gamma_{\tilde{s}}\left(\omega \right)\;d\omega }.$$

**Figure 8:**
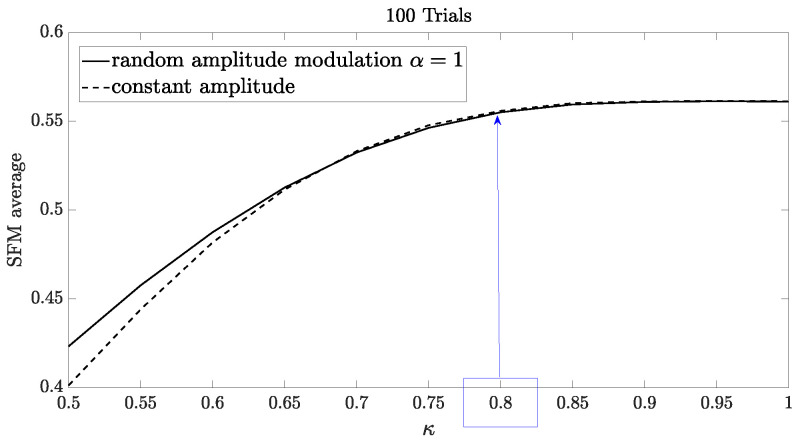
SFM average of APCN (κ≥0.5) waveforms: assuming Phase-Only randomness (constant amplitude) and random amplitude modulation (α=1).

From Figure 8, it is possible to observe that the SFM (calculated as the average of 100 independent trials) increases as κ increases, reaching an upper bound value when the phase factor is κ=0.8. Note also that the SFM average exhibits a similar behavior, assuming either random or constant amplitude. This observation suggests that the random phase contributes more significantly to the LPI/LPE characteristics.

The degree of randomness in a given stochastic waveform introduces an additional advantage to pulsed noise radars. Random signals are expected to present a low cross-correlation between pulses transmitted at different times. This feature enables the elimination of range ambiguities in pulse compression architectures.

A short-integration-time pulsed noise radar emits a train of noise-modulated electromagnetic pulses toward the target [2], specifically $N_{p}$ time-limited signals, separated in time by the Pulse Repetition Interval ($P_{RI}$). When we assume the presence of a single nonfluctuating scattering point moving at a range of $R_{0}$, at the time it starts being illuminated by the radar, we can express the complex envelope of the *i*-th received signal as [38]

(22) $${\tilde{r}}_{i}\left(t\right)=Ge^{-j4\pi \left(\frac{R_{0}+vT_{i}}{\lambda }\right)}\;e^{-j2\pi f_{D}t}\;{\tilde{s}}_{i}\left(t-T_{0}-\frac{2v}{c}T_{i}\right),$$

where *G* is a term that reflects the backscattering effects, channel fading, and the gains and distortions introduced by the receiver RF chain (assumed to be constant over the coherent processing interval), λ is the operating wavelength, and *v* is the target’s radial velocity, also assumed constant. Finally, $T_{0}$ in Equation (22) represents the time spent by the echo signal to return to the radar, which is given by $T_{0}=2R_{0}/c$ and $T_{i}=\left(i-1\right)P_{RI}$.

Range ambiguity arises when the scatter is far enough so that the *i*-th echo, relative to the reflection of the *i*-th transmitted pulse, arrives at the receptor after the transmission of the subsequent signal, $s_{i+1}\left(t\right)$, and, considering the pulse compression gain, has enough power to be detected. Let us again consider the simplified received signal model [21] for simplification and no loss of generality ${\tilde{r}}_{i}\left(t\right)=\tilde{s}\left(t-T_{0}\right)e^{-j2\pi f_{D}t}$, then, the expected value of the pulse compression output when range ambiguity is present is given by

(23) $$E\left[\tilde{y}{\left(\tau \right)}_{RA}\right]=E\left[\int_{-\infty }^{\infty }\left[{\tilde{s}}_{r_{i}}\left(t-T_{0}\right){\tilde{s}}_{c_{i}}\left(t-T_{0}\right)\;e^{-j2\pi f_{D}t}\right]\;{\tilde{s}}_{r_{i+1}}^{*}\left(t-\tau \right){\tilde{s}}_{c_{i+1}}^{*}\left(t-\tau \right)dt\right].$$

The stochastic nature of pure noise radar waveforms may contribute to the suppression of range ambiguities in target detection [39]. The cross-correlation process between the *i*-th replica and the *j*-th received pulse, where i≠j, plays a crucial role in radar systems employing random signals. The elimination of range ambiguity is directly contingent upon this process. It is no different for APCN signals. One can also evaluate range ambiguity suppression using the cross-correlation between the *i*-th and the *j*-th, transmitted pulses, i≠j, since it is possible to rewrite Equation (23) as

(24) $$E\left[{\tilde{y}}_{RA}\left(\tau \right)\right]=E\left[a_{i}\left(t\right)a_{i+1}\left(t-\tau \right)\right]E\left[e^{j\kappa \phi_{i}\left(t\right)}e^{j\kappa \phi_{i+1}\left(t-\tau \right)}\right]\;\chi_{{\tilde{s}}_{c}}\left(\tau -T_{0},f_{D}\right),$$

with $\left(T_{0}-\tau_{s}\leq \tau \leq T_{0}+\tau_{s}\right)$ and considering that ${\tilde{s}}_{c_{i}}\left(t\right)={\tilde{s}}_{c_{i+1}}\left(t\right)$.

Finally, after some mathematical manipulations, it can be shown that

(25) $$E\left[{\tilde{y}}_{RA}\left(\tau \right)\right]=\left(\alpha^{2}\frac{\pi }{2}\right)\left(\frac{1-\cos2\kappa \pi }{2\kappa^{2}\pi^{2}}\right)\;\chi_{{\tilde{s}}_{c}}\left(\tau -T_{0},f_{D}\right);\;\;\;\;T_{0}-\tau_{s}\leq \tau \leq T_{0}+\tau_{s}.$$

The attenuation in the pulse compression output of APCN signals introduced by the presence of the random component ${\tilde{s}}_{r}\left(t\right)$, when range ambiguous targets are present, is given by $H=\alpha^{2}\left[1-\cos(2\kappa \pi \right)]/4\kappa^{2}\pi$, the same introduced as an effect of the Doppler mismatch of moving targets (see Equation (5)). The same analysis can be performed for the Phase-Only APCN waveform, leading to

(26) $$E\left[{\tilde{y}}_{RA}^{PO}\left(\tau \right)\right]=\left(\frac{1-\cos2\kappa \pi }{2\kappa^{2}\pi^{2}}\right)\;\chi_{{\tilde{s}}_{c}}\left(\tau -T_{0},f_{D}\right);\;\;\;\;T_{0}-\tau_{s}\leq \tau \leq T_{0}+\tau_{s},$$

with an attenuation given by $D=\left[1-\cos(2\kappa \pi \right)]/2\kappa^{2}\pi^{2}$.

The primary objective of the radar system in surveillance applications is to provide the user with situational awareness of the operational scenario. That entails detecting uncooperative targets and determining their range and radial velocity in real-time. Contemporary digital systems utilize the Pulse Doppler technique, separable two-dimensional processing in the fast and slow-time dimensions, for that purpose [34].

In the fast-time dimension, the *i*-th single-pulse matched filter, denoted as ${\tilde{y}}_{i}\left(n\right)={\tilde{y}}_{i}{\left(t\right)}_{{|}_{t=n/f_{s}}}$ (see Figure 3) is performed. Next, the *k*-points Discrete Fourier Transform (DFT) of the slow-time data sequence is employed in each range bin [38]. The result is the well-known range-Doppler matrix, used as the input for the subsequent step of the detection process.

In the present analysis, we chose the Cell-Averaging (CA) CFAR detection technique to investigate the performance of APCN signals. We assume a single Signal of Interest (SOI) scenario and consider the predominant interference in the radar reception chain from thermal noise origin. Furthermore, employing a quadratic law detector, we set the probability of false alarm to $10^{-5}$. This detection process is first applied to the echo of the APCN waveform using the receiver system configuration detailed in [21]. Therefore, one must derive the matched filter output by correlating the receiver echo with the transmitted stochastic signal. Subsequently, the detection process assesses the corresponding filter output, as determined by Figure 3. Given the time and frequency sampling, we incorporate two “guard cells” on each side of the “cell under test”. We take this precaution to account for the potential occurrence of “straddle range-Doppler cells” [34].

Next, we discuss an experiment assuming a digital radar receiver. Below is an outline of the main parameters employed in the simulation for this experiment:

- Waveform wavelength: 0.0321 m;
- Waveform bandwidth: $\beta_{s}=30$ MHz;
- Waveform pulse width: $\tau_{s}=50\;\mu$s;
- Waveform pulse repetition interval: $\tau_{s}=500\;\mu$s;
- Fast-time sampling frequency: $f_{s}=125$ MHz;
- Pulse Repetition Frequency: PRF=2 kHz;
- Slow-time sampling frequency: 2 kHz;
- Number of received pulses: $N_{p}=27$;
- Number of DFT Doppler domain points: N=64;
- Target’s Doppler shift: $f_{D}=700$ Hz;
- Target’s range: $R_{0}=50$ km;
- Maximum radar unambiguous range: $Runam_{max}=75$ km.

**Figure 9:**
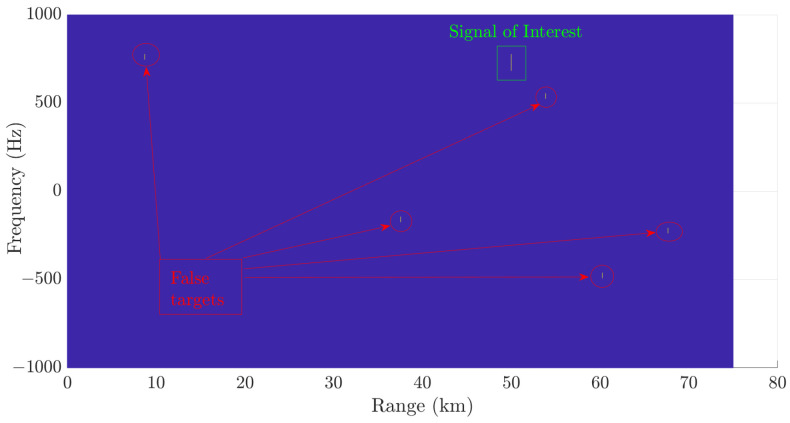
Detection process output considering the CA-CFAR technique implemented on the digital radar receiver proposed in [21].

Figure 9 displays the results of the detection process with the receiver system configuration detailed in [21]. In addition to successfully detecting the SOI highlighted in green, the five false targets depicted in red were also identified in a scenario with an SNR as low as −15 dB.

**Figure 10:**
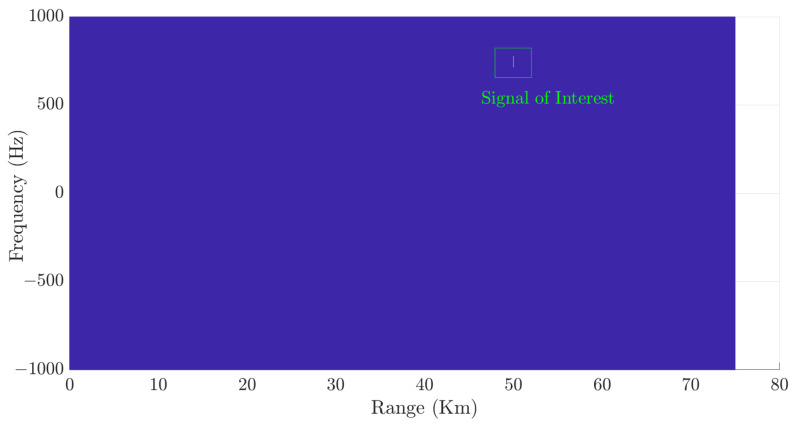
Detection process output considering the CA-CFAR technique applied to our proposed architecture.

Figure 10 shows the detected SOI when the configuration outlined in Figure 3 is employed. Note that this detection procedure eliminates false targets.

To demonstrate and validate the improvement in performance in detecting and estimating range and radial velocity, we conducted an experiment that consists of transmitting different APCN signals with randomness in amplitude (scale parameter α=1) and varied randomness in phase (scale parameter κ). The results illustrated in Figure 8 corroborate that phase randomness impacts the increase in APCN LPI/LPE characteristics most significantly. After applying the Pulse-Doppler processing, we generated a range-Doppler matrix, assuming a low SNR of −15 dB on the receiver’s end of the radar chain. Next, we applied the CA-CFAR detection technique to this dataset and calculated the average number of false targets. We conducted this analysis using Monte Carlo simulations comprising 100 independent trials. The procedure adheres to the recommended receiver configuration of [21] and the receiver configuration specifications provided in Figure 3.

**Figure 11:**
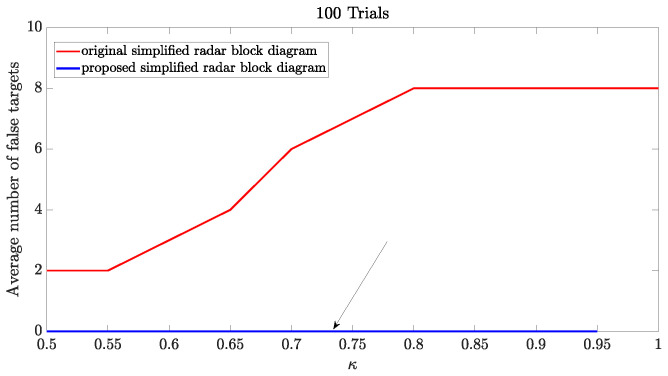
APCN waveform (κ≥0.5 and α=1) detection performance: average number of false targets.

Figure 11 illustrates the results of an experiment in which we increased the waveform randomness while maintaining the SNR level low in the radar receiver chain. Notably, using the configuration detailed in [21] resulted in a substantial increase in the average count of false targets. Therefore, this configuration significantly compromises the radar’s detection and estimation capabilities despite enhancing its LPI/LPE characteristics. Specifically, when the variable κ reaches a value of 0.8, the average count of false alarms escalates to eight. This elevated count remains consistent regardless of increments in the value of κ. This behavior relates to the maximum value of the SFM average observed in Figure 8, which reaches an upper bound when the phase factor is κ=0.8.

However, when we applied our proposed architecture detailed in Figure 3, it maintained the crucial LPI/LPE capability by preserving the transmission waveform. Moreover, as we explored phase scale factors up to κ=0.95, we eliminated false target occurrences, reducing them notably to zero, as visually indicated by the black arrow in Figure 11. However, the waveform lost its Doppler tolerance capacity at the uppermost limit of randomness, namely κ=1. Consequently, correlating the received echo from the APCN waveform with its deterministic component became unfeasible.

It is clear from the previous derivations and analysis that proper selection of the parameters governing the random components of the APCN transmitted signal is relevant for the overall system performance. The greater the value of κ, the more random the waveform. That leads to a higher Spectral Flatness Measure, improving its range ambiguity suppression. On the other hand, high values of κ lead to a waveform with less spectral efficiency (low power within the desired bandwidth) and less Doppler tolerance. Using a stochastic signal to modulate the transmit waveform in amplitude also increases its degree of randomness, which enhances the trade-offs discussed above. Additionally, the random component in amplitude also deteriorates the system performance concerning power efficiency, increasing the transmit signal’s PAPR. That needs to be considered, especially in long-range applications.

In Section 3, we examine APCN waveforms from the point of view of a passive intercept-receiver system and propose, analyze, and discuss a methodology to detect and extract the characteristics of this noisy waveform automatically. These waveforms are recognized for their LPI/LPE attributes as detailed in [3,20].

## 3. The Proposed Metodology for Identifying APCN Signals

In this section, we investigate the performance of the APCN waveform in an electronic warfare context. We first address the modeling of a digital superheterodyne ESM receiver system. We consider interception, A/D conversion, and digital processing operations performed on the analog SOI. We introduce a specific time-frequency transform technique to analyze radar signals deemed as LPI/LPE [11,23]. Then, we outline techniques used to extract signal characteristics and evaluate the performance of the proposed methodology in estimating the radar parameters of the SOI in an EW scenario.

The primary objective of an ESM system is to identify emitting sources. The ESM system’s digital processing chain extracts intrapulse and interpulse parameters from the intercepted waveform. Examples of intrapulse parameters are pulse width ($\tau_{s}$), operating frequency ($f_{0}$), and bandwidth ($\beta_{s}$), whereas for interpulse we have the Pulse Repetition Interval ($P_{RI}$). The way to determine the Pulse Repetition Interval is by estimating the arrival time ($T_{e}$) between successive intercepted pulses [40]. This information is intrinsic to the signal’s identity. As for the amplitude of the received signal, it relies, in part, on the distance between the radar and the ESM system since measuring the amplitude is challenging due to deleterious effects, such as multipath that may lead to constructive interference within pulses [40].

Commonly, an ESM system has a listening time (Δt) longer than the radar’s pulse repetition interval, enabling parameter estimation based on multiple intercepted pulses. Thus, the intercepted RF signal can be expressed as

(27) $$r_{e}^{RF}\left(t\right)\;=\;\sum_{i\;=\;1}^{Np_{e}}Q\;s_{i}^{RF}\left(t-T_{e}-\left(i-1\right)P_{RI}\right),$$

where $s_{i}^{RF}\left(t\right)$ is the *i*-th transmitted pulse one-way Doppler shifted by the radial relative velocity. Moreover, *Q* accounts for gains and attenuation, and $N_{p_{e}}$ is the number of intercepted pulses.

Typically, six pulses are needed to allow parameter extraction [40]. The received signal is routed to the RF chain and contaminated with thermal noise w(t). Therefore, the actual signal at the output of the RF module is given by

(28) $$x\left(t\right)=r_{e}\left(t\right)+w\left(t\right)=\sum_{i=1}^{N_{p_{e}}}V\;s_{i}\left(t-T_{e}-\left(i-1\right)P_{RI}\right)+w\left(t\right),$$

where $s_{i}\left(t\right)$, in noise radars, are sample functions of the stochastic process that characterizes the transmitted random signal assumed to be statistically independent of the thermal noise. Moreover, *V* accounts for gains and attenuation introduced by the RF chain cascaded to the gains and attenuation of the intercepted signal. We assume real-valued and time-invariant quantities throughout this work.

**Figure 12:**
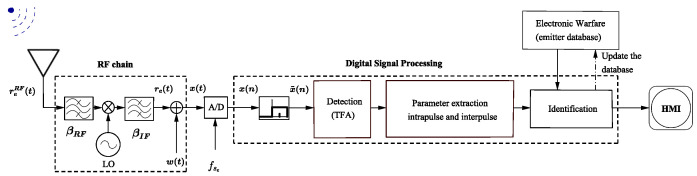
ESM superheterodyne receiver: simplified block diagram incorporating digital technology.

Since we employ discrete-time analysis, we denote ${x\left(n\right)=x\left(t\right)|}_{t=n/fs_{e}}$, assuming a digital ESM system sampling frequency of $f_{s_{e}}$. Additionally, the TFA of the intercepted radar signal adopts its analytical form [11] and is given by

(29) $$\tilde{x}\left(n\right)\;=\;x\left(n\right)+jH\left\{x(n)\right\},$$

where H denotes the Hilbert transform. Figure 12 illustrates the simplified diagram of an ESM superheterodyne digital receiver [11], outlining the process that starts with the reception of the analog signal up to the display of the extracted information in a Human–Machine Interface (HMI). The diagram excludes the pulse deinterleaving step, focusing the analysis on individual waveforms.

In numerous EW scenarios, the nature of a received radar signal often deals with time and frequency information and, therefore, requires time-frequency analysis techniques to effectively characterize the non-stationary behavior exhibited by the signal [23,41]. Several variations of these techniques are available in the literature. We use the Short-Time Fourier Transform (STFT) in this work, with no loss of generality.

The discrete version of the STFT of signal $\tilde{x}\left(n\right)$ is defined as

(30) $$S_{F}\left(k,m\right)=\sum_{n\;=\;0}^{N_{e}-1}\tilde{x}\left(n+m\;R\right)\;g\left(n\right)\;e^{-j\left(\frac{2\pi k}{N_{e}}\right)n};\;0\leq k\leq N_{e}-1,$$

where $2\pi k/N_{e}$ is the *k*-th discrete frequency bin, *m* represents the *m*-th discrete tile in time, $N_{e}$ is the number of points of the FFT, equal to the window size, and *R* is the hop size (with an overlapping of $N_{e}-R$ samples in this case). Moreover, g(n) is a window of size $N_{e}$, that is, g(n)=0 outside the interval $0\leq n\leq N_{e}-1$. In this work, we chose the Hamming window, widely used in EW systems [11,34,41]. In most applications that involve STFT, the interest is in the magnitude response, with a focus on the short-time quadratic magnitude $|S_{F}{\left(k,m\right)|}^{2}$, representing the short-time energy spectral density, and usually displayed as a function of time and frequency in the form of a spectrogram [42].

From the computational implementation of Equation (30), we obtain the $N_{e}\;\times \;M$ matrix E of time-frequency distribution, given by

(31) $$E={\left[\begin{array}{ccc} \vdots & \vdots & \vdots \\ |S_{F}{\left(k,1\right)|}^{2} & \cdots & |S_{F}{\left(k,M\right)|}^{2}\\ \vdots & \vdots & \vdots \end{array}\right]}_{N_{e}\times M},$$

where *k* corresponds to *k*-th frequency bin, $N_{e}$ is the total number of bins, and *M* is the total number of tiles in time. The determination of overlap, crucial for achieving resolution between fixed tile and frequency bin quantities, is computed according to [43]

(32) $$L=\left\lceil \frac{MN_{e}-N_{s}}{M-1}\right\rceil ,$$

where “⌈ ⌉” is the round-to-nearest integer operator and $N_{s}$ is the number of samples.

**Figure 13:**
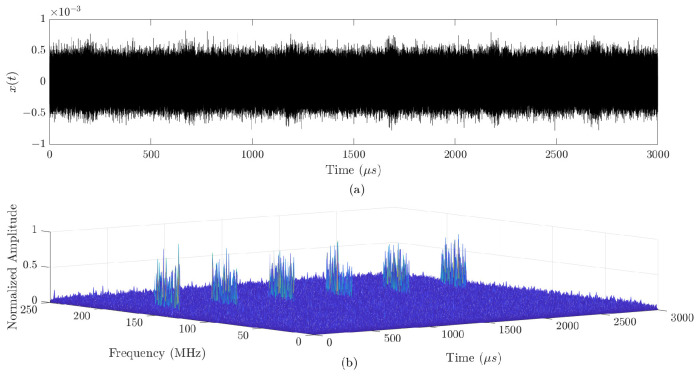
Intercepted signal x(t): (**a**) time representation; (**b**) time-frequency representation. In this figure, the x-axis is represented by “Time (μs)” and the y-axis is represented by “Frequency (MHz)”.

Displayed in Figure 13 are the time “(a)” and time-frequency “(b)” representations of an intercepted signal, derived from six pulses of an APCN waveform (κ=0.5 and α=1). It is important to note that while representation “(a)” highlights time-related features, the time-frequency display in “(b)” provides valuable insights into the energy carried by the SOI. These depictions assume an SNR of −10 dB at the passive intercept-receiver input.

The subsequent stage after the TFA in the block diagram depicted in Figure 12 involves parameter extraction. Matrix E described by Equation (31) furnishes details regarding the energy the intercepted signal carries, enabling us to visualize the time-frequency plane as a 2D image. Consequently, it is feasible and natural to use image processing techniques to extract parameters related to the SOI. In the present work, we use the Hough transform for detecting geometric shapes such as lines in a binary image [44]. The way to represent a line equation in the Hough space is as follows [45]:

(33) ρ=xcos(ψ)+ysin(ψ),

where ρ is the distance between the line and the origin of the Cartesian system and ψ is the angle between the axis *x* and the segment perpendicular to the line.

**Figure 14:**
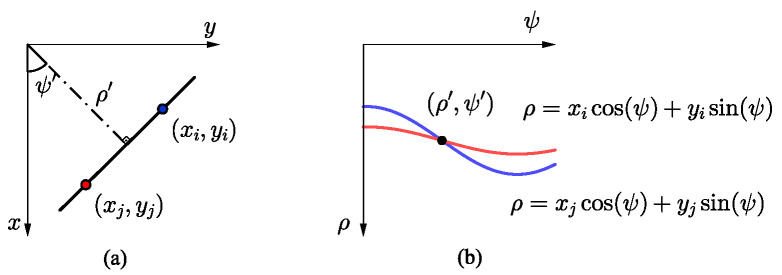
Representation of a line: (**a**) Cartesian space; (**b**) Hough space.

Figure 14 illustrates the process of estimating a line in Cartesian space (a) from the Hough space (b), with the parameters $\psi^{\prime }$ and $\rho^{\prime }$ determined by the intersection of the two sinusoids within the Hough space.

The computational implementation of the Hough transform [46] yields a structured array represented as $\left[H;\psi ;\rho \right]$, where H denotes the histogram amplitude matrix, with each of its elements standing for the number of increments within each cell of the quantized Hough space. Additionally, ψ represents the slopes vector, while ρ is the distance vector.

The peaks in H are then obtained and stored in a matrix P of order $n_{p}\times 2$ whose form is given by

(34) $$P=\left[\rho^{*}\psi^{*}\right]=\left[\begin{array}{cc} \rho_{1} & \psi_{1}\\ \rho_{2} & \psi_{2}\\ \vdots & \vdots \\ \rho_{n_{p}} & \psi_{n_{p}} \end{array}\right],$$

where $n_{p}$ represents the desired number of peak estimates, assumed to be the minimum number of pulses $N_{p_{e}}$ required to enable the parameter extraction output. Finally, the detection of lines in the image space (matrix BW) is performed, leading to L, a *restructured array* of the form $\left[L_{1};\;L_{2}\right]$ where

(35) $$L_{1}=\left[\begin{array}{cc} x_{1}{|}_{P_{1}} & y_{1}{|}_{P_{1}}\\ \vdots & \vdots \\ x_{1}{|}_{P_{i}} & y_{1}{|}_{P_{i}}\\ \vdots & \vdots \\ x_{1}{|}_{P_{N_{p_{e}}}} & y_{1}{|}_{P_{N_{p_{e}}}} \end{array}\right],$$

with $x_{1}{|}_{P_{i}}$ and $y_{1}{|}_{P_{i}}$ being the *i*-th ordered pair matrix referring to the beginning of the *i*-th detect line, and

(36) $$L_{2}=\left[\begin{array}{cc} x_{2}{|}_{P_{1}} & y_{2}{|}_{P_{1}}\\ \vdots & \vdots \\ x_{2}{|}_{P_{i}} & y_{2}{|}_{P_{i}}\\ \vdots & \vdots \\ x_{2}{|}_{P_{N_{p_{e}}}} & y_{2}{|}_{P_{N_{p_{e}}}} \end{array}\right],$$

is the matrix representing the ordered pairs at the end of the *i*-th detected line.

The intrapulse and interpulse parameters of the SOI are then estimated, with the estimated bandwidth given by

(37) $${\hat{\beta }}_{s}=\frac{1}{N_{p_{e}}}\left[\sum_{i=1}^{N_{p_{e}}}\left(y_{2}{{|}_{P_{i}}-y_{1}|}_{P_{i}}\right)\frac{f_{s_{e}}}{2N_{e}}\right].$$

The SOI operating frequency ($f_{0}$) can be estimated as

(38) $$\hat{f_{c}}=\frac{1}{N_{p_{e}}}\left[\sum_{i=1}^{N_{p_{e}}}\left(y_{1}{|}_{P_{i}}+0.5\;\left(y_{2}|P_{i}-y_{1}|P_{i}\right)\right)\frac{f_{s_{e}}}{2N_{e}}\right].$$

Therefore, $\hat{f_{0}}=\hat{f_{c}}+f_{LO}$, where $f_{LO}$ is the ESM system Local-Oscillator (LO) frequency. The estimated intercepted signal time duration is calculated as

(39) $$\hat{\tau_{s}}=\frac{1}{N_{p_{e}}}\left[\sum_{i=1}^{N_{p_{e}}}\left(x_{2}|P_{i}-x_{1}|P_{i}\right)\frac{\Delta t}{M}\right].$$

Finally, to estimate the radar $P_{RI}$, it is necessary to measure the difference between the arrival times of $N_{p_{e}}$ successive pulses in such a way that

(40) $${\hat{P}}_{RI}=\frac{P_{RI}{|}_{P_{\left(21\right)}}+P_{RI}{|}_{P_{\left(32\right)}}+\cdots +P_{RI}{{|}_{P_{\left(i(i-1)\right)}}+\cdots +P_{RI}|}_{P_{\left(N_{p_{e}}\left(N_{p_{e}}-1\right)\right)}}}{N_{p_{e}}-1},$$

where $P_{RI}{|}_{P_{\left(i(i-1)\right)}}=\left(x_{1}{{|}_{P_{i}}-x_{1}|}_{P_{\left(i-1)\right)}}\right)\frac{\Delta t}{M}$ is the *i*-th measure pulse repetition interval between two successive lines detected.

**Figure 15:**
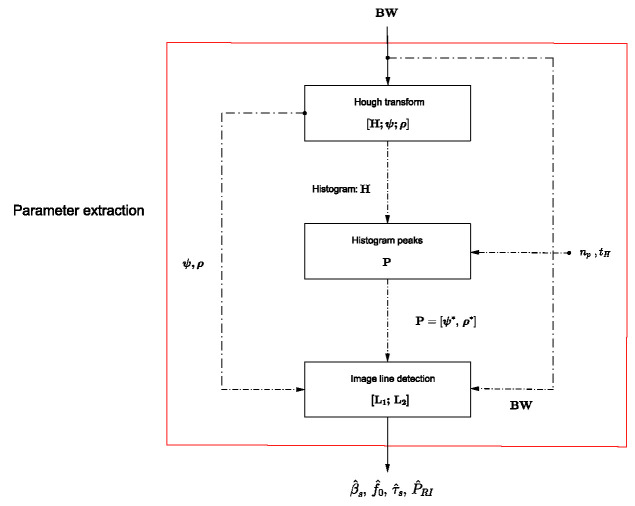
Block diagram showing the approach for extracting parameters from the APCN noise radar waveform.

The block diagram in Figure 15 illustrates the methodology proposed in this study for parameters extraction from the APCN noise radar waveform. In this diagram, solid black lines represent the input and output of the block diagram, while dashed black lines indicate intermediate inputs and outputs necessary for preprocessing.

In the final stage of the architecture outlined in Figure 12, emphasis is placed on identifying the source emitter. In the digital processing realm of an ESM system, a predefined set of mean parameters ${\left[{\hat{\beta }}_{s}\;{\hat{f}}_{0}\;{\hat{\tau }}_{s}\;{\hat{P}}_{RI}\right]}^{T}$, referred to as a *fingerprint*, can aid in the identification phase of the radar model [1,11,40]. These parameters offer a degree of tolerance and facilitate the identification of the emitting source. In cases where the intercepted signal fails to correlate with an existing emitter in the EW database, we add the new signal to the database for future recognition.

Subsequent sections delve into the proposed methodology, utilizing a numerical example to detail the process of extracting signal information to construct a waveform *fingerprint*.

### 3.1. Numerical Example

**Figure 16:**
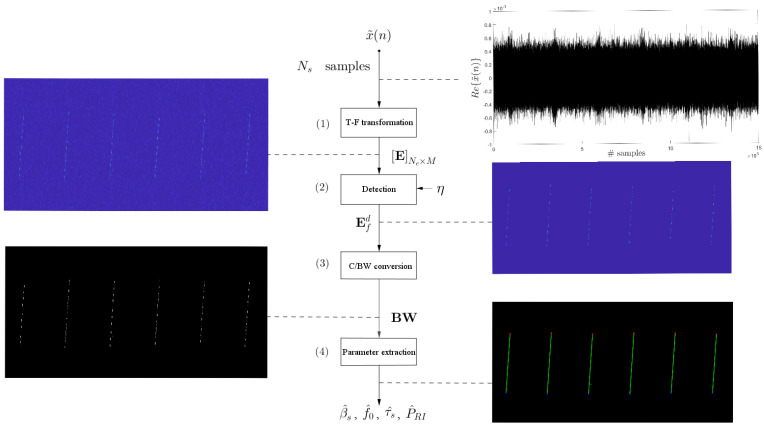
Block diagram illustrating a proposed methodology utilizing image processing techniques. Parameters to be extracted include bandwidth, operating frequency, pulse width, and pulse repetition interval.

The block diagram of Figure 16 outlines the proposed methodology. It is designed to aid in identifying APCN signals through image processing, thereby enabling the identification of emitting sources transmitting this type of waveform in an EW scenario. The synthesized scenario is based on Figure 12 and the main parameters used in the simulations are:

- Waveform bandwidth: $\beta_{s}=30$ MHz;
- Waveform pulse width: $\tau_{s}=50\;\mu$s;
- Waveform pulse repetition interval: $P_{RI}=500\;\mu$s;
- ESM system sampling Frequency: $f_{s_{e}}=500$ MHz;
- ESM system listening time: Δt=3000μs;
- ESM system local oscillator frequency: $f_{LO}=9.2$ GHz;
- Number of pulses intercepted by the ESM system: $N_{p_{e}}=6$;
- Radar center frequency: $f_{c}=160$ MHz.

It is noteworthy to emphasize that, in [20], the utilization of an APCN waveform with specific parameters (κ=0.5 and α=1) and a designed bandwidth of 30 MHz was identified as potentially posing challenges to the intercept–receiver system in a communication channel where thermal noise was present. Acknowledging this insight from the referenced work, our investigation aligns with this perspective, thus adopting a bandwidth of $\beta_{s}=30$ MHz for our analysis.

The proposed methodology to analyze this noise radar signal comprises four steps (as seen in Figure 16) and are described in the following.

1. In Step 1, time-frequency (T-F) transformation, we obtain matrix E using Equation (31). To address the uncertainty principle [42], we use a window g(n) of size $N_{e}=1024$ samples, corresponding to 2.048 μs. This choice aims to balance local signal analysis and stationary conditions, facilitating FFT applications while ensuring an adequate balance of time and frequency resolutions. The analytical nature of signal $\tilde{x}\left(n\right)$ allows for 512 frequency bins within the range $0\leq f\leq f_{s_{e}}/2$, corresponding to the positive half of the spectrum 0≤ω≤π, given the window size. Simultaneously, we set the number of STFT tiles to M=1024, with an overlap of 536 samples at each hop. As illustrated in Figure 16, this transformation showcases the signal’s shift from the time domain to its time-frequency representation. Although the radar signal’s intent is discernible amidst system thermal noise, preprocessing remains necessary for automatic and accurate characteristic extraction.
2. Step 2 performs detection in the T-F plane. For this purpose, we define a threshold η as
  (41) $$\eta =-\left[\bar{e}\;\ln\left(P_{fa}\right)\right],$$
  with
  (42) $$e=\underset{\mathrm{Mean}\;\mathrm{vector}\;\mathrm{in}\;\mathrm{matrix}\;\mathrm{rows}}{\underset{︸}{{\left[\;\bar{E_{f}\left(:\;,1\right)}\;\;\;\;\bar{E_{f}\left(:\;,2\right)}\;\;\;\;\cdots \;\;\;\;\bar{E_{f}\left(:\;,M\right)}\;\right]}^{T}}}\;\mathrm{and}\;\bar{e}=\frac{1}{M}\;\sum_{m=1}^{M}\bar{E_{f}\left(:\;,m\right)}\;,$$
  where the desired probability of false alarm is $P_{fa}=10^{-5}$. After the detection process, matrix $E_{f}^{d}$ is obtained as the output, as illustrated in Figure 16.
3. Conversion from grayscale (C) to black-white (BW): $E_{f}^{d}$ is converted from grayscale to black-and-white [47] to obtain the matrix BW, and the signal’s amplitude information is encoded into binary values.
4. Parameter extraction step: as previously mentioned, the proposed approach for extracting information from the APCN waveform relies on the Hough transform. Due to its deterministic component, and according to [3,22], the bandwidth $\beta_{s}$ can be considered the same as that of its linear chirp component, i.e., $\beta_{s_{c}}$. The number of input peaks, assumed to be the minimum number of pulses to allow for parameter extraction, was considered $n_{p}=6$ [40]. Moreover, we fixed the threshold $t_{H}$ at $0.5\max\left[H\right]$, which is the default minimum value for identifying a peak.

**Figure 17:**
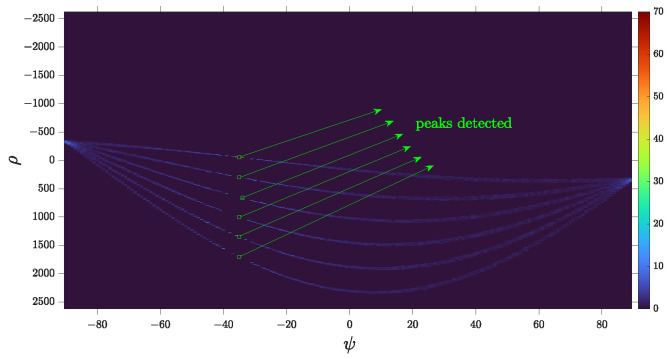
Peaks detected from Hough’s histogram $\left[H\right]$.

**Figure 18:**
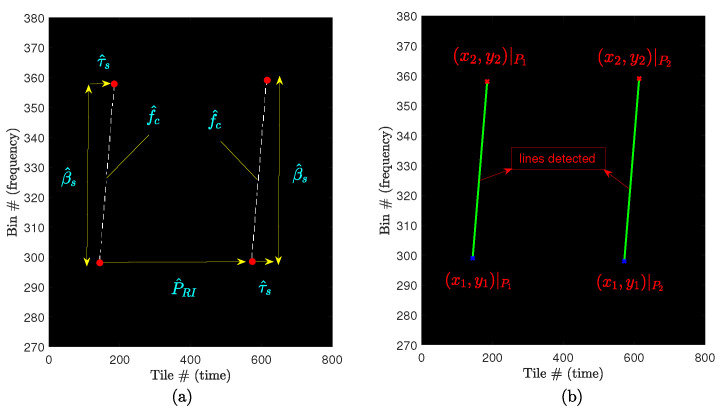
Detected lines. For better observation, the image has been zoomed in on the first two intercepted pulses (**a**) desired parameters estimation; (**b**) Cartesian lines detected. In both figures “#” stands for the number of the Bin or Tile, respectively.

By defining a threshold $t_{H}$ and applying it to the Hough space matrix H, along with knowing the desired minimum number of peaks $n_{p}$, we can obtain the matrix P as denoted by Equation (34). Figure 17 illustrates the detected peaks stored in P. Subsequently, using $P=\left[\rho^{*}\;\psi^{*}\right]$, the conversion from Hough space to Cartesian space [45] was performed based on the parameter relationship in Equation (33), resulting in the detection of lines $\left[L_{1};\;L_{2}\right]$. Figure 18b illustrates some of the lines detected in Cartesian space.

Finally, the intrapulse and interpulse parameters are estimated as depicted in the detailed extraction block diagram (Figure 15). This process establishes a connection between the desired information illustrated in Figure 18a and the information obtained in the Cartesian space through the Hough space (Figure 18b).

### 3.2. Performance Assessment

Several studies in the literature aim to establish performance benchmarks for ESM systems [29,48,49], yet a universally accepted standard for ESM development remains elusive [30]. As previously mentioned, tolerances may correlate a particular set of estimated parameters to a specific emitter, and their significance in the overall ESM performance is crucial [40]. For instance, in [29], the assumed tolerances are ±1 MHz for operating frequency and bandwidth and ±1 μs for the modulation period. From this perspective, one presumes that the probability of an ESM detecting a radar signal ($P_{d_{e}}$) is directly linked to the accuracy/precision of its parameter estimation by such a system, making it an evaluation metric. As an alternative, the authors in [23,29,31] consider the percentage relative error to evaluate the efficiency of their proposed methodologies for radar parameter extraction of deterministic radar signals considered LPI/LPE.

**Figure 19:**
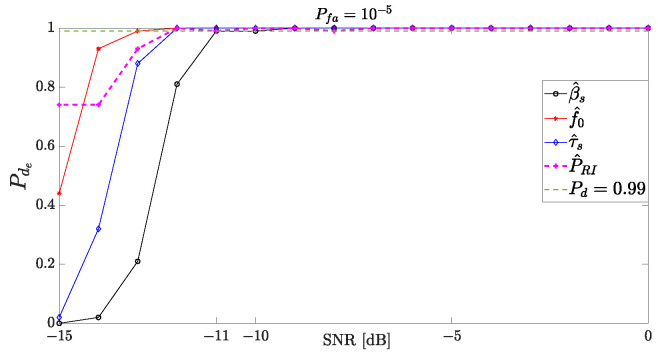
Probability of detection APCN waveform parameters (κ=0.5 and α=1) considering 100 independents trials.

In this assessment, we start with $P_{d_{e}}$ of the ESM system employing the proposed methodology to identify APCN signals through a Monte Carlo simulation, assuming 100 independent trials. For this purpose, we record a detection when the tolerance between the actual and estimated parameters falls below a certain threshold: ±2 MHz for $\beta_{s}$, ±5 MHz for $f_{0}$, ±5 μs for $\tau_{s}$ and ±25 μs for $P_{RI}$. These tolerances were derived from the information in [29,40]. Figure 19 presents the obtained ESM probability of detection, $P_{d_{e}}$, of an APCN signal. One can see that the detection performance remains above 99% for SNR levels considered low [23,29,30,31], i.e., less than −10 dB, for both intrapulse as well as interpulse radar parameters. Performance is degraded for SNR less than −11 dB.

We also evaluated the accuracy and precision of the random variables characterizing the proposed estimators. Accuracy, represented by the bias of an estimator $\hat{\Theta }$ for a parameter Θ [11,34], is defined as the expected value of the difference between the mean of the estimate and the actual parameter value

(43) $$B_{\hat{\Theta }}\left(\Theta \right)=E\left[\hat{\Theta }\right]-\Theta .$$

Precision, on the other hand, is the standard deviation of the estimate

(44) $$\sigma_{\hat{\Theta }}\left(\Theta \right)=E\left[\sqrt{{\left(\hat{\Theta }-E\left[\hat{\Theta }\right]\right)}^{2}}\right].$$

**Figure 20:**
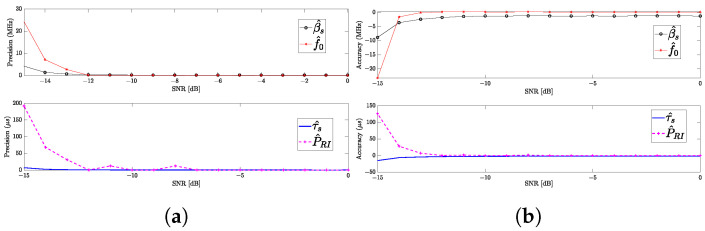
APCN (κ=0.5 and α=1) waveform parameters estimation performance: (**a**) Precision as a function of the SNR; (**b**) Accuracy as a function of the SNR.

Figure 20a and Figure 20b, respectively, depict the precision and accuracy of the proposed estimators across varying SNR levels in an ESM system employing the methodology to identify APCN signals. The estimators exhibit high precision, implied by the low standard deviation of the random variables, up to an SNR of −12 dB. However, beyond this threshold, a notable decline in precision is observed. Additionally, while the proposed $f_{0}$ estimator maintains high accuracy up to an SNR of −12 dB before exhibiting bias, the proposed bandwidth estimator displays a slight bias (approximately 1 MHz) independent of the SNR in the passive intercept–receiver chain. Nonetheless, the estimators for intrapulse and interpulse temporal parameters demonstrate high accuracy up to an SNR of −12 dB before showing signs of bias.

Thus, from the analyzed perspectives, a digital intercept receiver that employs the automatic parameter extraction approach proposed in the present work can detect the APCN noise radar signal, with κ=0.5 and α=1, and explore it, inhibiting this waveform from being claimed as either LPI or LPE.

**Figure 21:**
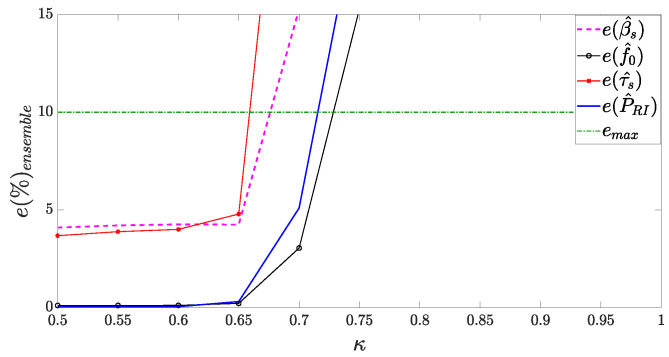
APCN parameter intrapulse and interpulse estimation with different values of κ, considering α=1.

Lastly, Figure 21 presents the assessment metric based on percentage error, defined as

(45) $$e\left(\%\right)=\left|\frac{\mathrm{actual}\;\mathrm{value}-\mathrm{estimated}\;\mathrm{value}}{\mathrm{actual}\;\mathrm{value}}\right|\times 100,$$

wherein the mean percentage relative error $e{\left(\%\right)}_{ensemble}$ is derived for observation in the experiment ensemble. As per [1,31,50], a margin of up to 10% in parameter estimation error can be deemed acceptable in the context of ESM equipment.

We have used that metric to evaluate the performance of the proposed methodology for APCN signals generated with different values of κ, assuming α=1. Moreover, we have considered a fixed SNR of −3 dB in the receiver chain. As we can see, the proposed methodology managed to estimate all parameters within an acceptable error limit for APCN waveforms that employ κ<0.7 when the signal becomes too noisy, at the expense of spectral confinement and Doppler tolerance, as previously mentioned.

## 4. Conclusions

This paper investigated the performance of the Doppler-tolerant Advanced Pulse Compression Noise waveform radar in surveillance applications. We analyzed its performance as an LPI/LPE signal under the framework of a proposed detection/information extraction method. From the perspective of a radar system, we showed an expression of the narrowband ambiguity function to assess its Doppler tolerance capacity. The analysis revealed an anomaly inherent to the waveform that can jeopardize detecting slow-moving targets in surveillance applications. Thus, we proposed a novel configuration for a digital radar receiver to address this issue. The proposed solution involves correlating the received signal with the deterministic component of the APCN waveform instead of relying on the transmit signal’s replica in a pulse compression architecture. This approach eliminates the anomaly and improves the accuracy and reliability of slow-moving target detection within noisy environments at the expense of an additional attenuation of the resulting signal. Closed-form expressions characterizing the pulse compression output in such architecture were also derived.

Moreover, we showed that the meticulous selection of parameters governing the random components in the APCN transmit signal emerges as a pivotal factor influencing overall system performance. In particular, the scale parameter associated with phase randomness assumes a critical role: larger values yield a more random waveform characterized by a higher Spectral Flatness Measure, thereby enhancing range ambiguity suppression. However, this improvement is offset by reduced spectral efficiency, as higher scale parameters lead to lower power within the desired bandwidth and decreased Doppler tolerance. Furthermore, introducing a stochastic signal to modulate the transmit waveform’s amplitude intensifies its randomness, exacerbating these trade-offs. Additionally, the inclusion of a random component in amplitude results in a deterioration in system performance concerning power efficiency, as evidenced by the increased Peak-to-Average Power Ratio in the transmit signal. These considerations are particularly pertinent in long-range applications and necessitate careful deliberation in designing and optimizing APCN-based systems.

Regarding an intercept–receiver system point of view, a system with digital processing was modeled assuming a plausible number of intercepted pulses, according to the recent literature. We then proposed a candidate method to use in an ESM system for APCN noise waveform detection and parameter extraction. We employed a time-frequency transform to accurately extract the radar parameters since interception and exploitation of signals considered LPI/LPE requires sophisticated receivers that use time-frequency signal processing. The transformation made it possible to detect the radar signal immersed in thermal noise. We assumed the nonexistence of any replica of the intercepted signal, as the sample functions of noise radar are theoretically uncorrelated with each other, and the incoming signal parameters are unknown.

The proposed methodology parameter extraction was based on image processing techniques generated by the time-frequency transform. We described each step of the developed methodology to finally generate a *fingerprint* to assist in identifying the emitting source. We evaluated the intercept receiver performance based on the probability of such an ELINT system detecting an APCN radar signal, considering LPI/LPE as a function of the signal-to-noise ratio of the ELINT system. Results showed a probability of detection close to 1 for SNRs less than −10 dB. We also evaluated the accuracy and precision of the random variables characterizing the APCN estimated parameters (bandwidth, operating frequency, time duration, and pulse repetition interval) as a function of the SNR. Results also showed that the proposed ELINT system performed well in estimating such parameters in scenarios with SNR less than −10 dB. Finally, we concluded that defining a radar as LPI and LPE, or either, necessarily involves defining the corresponding intercept–receiver system.

## Data Availability

Data are contained within the article.
